# Therapeutic Notes

**Published:** 1899-09-09

**Authors:** 


### THERAPEUTIC NOTES.

### A MOUTH WASH.

MR. JOHN FITZGERALD, in his work on "Pyorrhœa Alveolaris," gives the following prescription for a mouth wash to be used night and morning:—

℞. Glyc. acid. carbol., fl. dr. 4.
Glyc. acid. boric., fl. dr. 4.
Potassæ chloratis, dr. 2.
Euthymol, dr. 4.
Aqua anisi.
Aqua menth. pip., āā ad. fl. oz. 8.

